# Chestnut Honey and Bacteriophage Application to Control *Pseudomonas aeruginosa and Escherichia coli* Biofilms: Evaluation in an *ex vivo* Wound Model

**DOI:** 10.3389/fmicb.2018.01725

**Published:** 2018-07-31

**Authors:** Ana Oliveira, Jéssica C. Sousa, Ana C. Silva, Luís D. R. Melo, Sanna Sillankorva

**Affiliations:** Centre of Biological Engineering, Laboratório de Investigação em Biofilmes Rosário Oliveira, University of Minho, Braga, Portugal

**Keywords:** *ex vivo*, *in vitro*, biofilms, dual-species, *P. aeruginosa*, *E. coli*

## Abstract

Chronic skin wounds represent a major burn both economically and socially. *Pseudomonas aeruginosa* and *Escherichia coli* are among the most common colonizers of infected wounds and are prolific biofilm formers. Biofilms are a major problem in infections due to their increasingly difficult control and eradication, and tolerance to multiple prescribed drugs. As so, alternative methods are necessary. Bacteriophages (phages) and honey are both seen as a promising approach for biofilm related infections. Phages have specificity toward a bacterial genus, species or even strain, self-replicating nature, and avoid dysbiosis. Honey has gained acknowledgment due to its antibacterial, antioxidant and anti-inflammatory and wound healing properties. In this work, the effect of *E. coli* and *P. aeruginosa* phages vB_EcoS_CEB_EC3a and vB_PaeP_PAO1-D and chestnut honey, alone and combined, were tested using *in vitro* (polystyrene) and *ex vivo* (porcine skin) models and against mono and dual-species biofilms of these bacteria. In general, colonization was higher in the porcine skins and the presence of a second microorganism in a consortium of species did not affect the effectiveness of the treatments. The antibacterial effect of combined therapy against dual-species biofilms led to bacterial reductions that were greater for biofilms formed on polystyrene than on skin. Monospecies biofilms of *E. coli* were better destroyed with phages and honey than *P. aeruginosa* monospecies biofilms. Overall, the combined phage-honey formulations resulted in higher efficacies possibly due to honey's capacity to damage the bacterial cell membrane and also to its ability to penetrate the biofilm matrix, promoting and enhancing the subsequent phage infection.

## Introduction

Chronic wounds are defined as wounds which failed the sequential reparative process responsible to repair the anatomic and functional integrity of the damaged tissue in a period of 4-8 weeks (Lazarus et al., 1994; Mustoe et al., 2006). These wounds lead to considerable morbidity and high costs associated with treatment, which represents an increasing burden on public and health systems worldwide.

In a chronic wound, bacterial growth occurs in biofilms, sessile communities organized in a three-dimensional structure, embedded in a self-produced matrix containing extracellular polymeric substances (EPS) such as polysaccharides, proteins, extracellular DNA, membrane vesicles, and other polymers. Biofilms are a protected mode of growth that allows bacteria to survive in hostile environments, presenting an altered growth rate (Baillie and Douglas, 1998) and gene expression (Whiteley et al., 2001), and an increased tolerance to antimicrobials (Fux et al., 2005), when comparing to their planktonic equivalents. Within a chronic wound, the biofilm tolerance to several antibiotics and host defenses (Flemming and Wingender, 2010) is promoted by numerous factors. The biofilm matrix offers structural stability, acting as a diffusional barrier both to antibiotics (Billings et al., 2013) and to host defenses (Jensen et al., 2007). Besides, extracellular DNA can be easily exchanged among bacteria allowing the transference of genes responsible by protective behaviors against external molecules (Chiang et al., 2013). For example, efflux pumps have been identified in several biofilm forming pathogens, such as *E. coli* (Ito et al., 2009), *P. aeruginosa* (Zhang and Mah, 2008) and *S. aureus* (Ding et al., 2008) and the production of antibiotic degrading-enzymes, such as β-lactamase, was identified in biofilm forming strains (Hengzhuang et al., 2013).

Bacteriophages (phages) are highly specific viruses that infect and replicate within bacteria. Phage attachment to a host cell occurs after specific recognition of complementary receptors on the bacterial cell surface (Weinbauer, 2004). The great increase of multi-drug resistant microorganisms has revitalized the interest in using phages as an effective alternative to antimicrobial therapy, including for wound healing (Pirnay et al., 2011).

Honey is a viscous solution derived from nectar gathered and modified by honeybee. It is composed by ~31.3% glucose, 38.2% fructose, 1% sucrose and 17% water, and in minor quantity by organic acids, proteins, amino acids, vitamins, minerals and enzymes (Bogdanov et al., 2008). The use of honey in wounds was firstly documented by the ancient Egyptians 4,000 years ago and it has been used for this purpose since ancient times, by Romans, Greeks, and Chinese (Sato and Miyata, 2000). Antimicrobial properties of honey are associated with a combination of factors as high osmolarity, low availability of water (Molan, 1992), production of hydrogen peroxide [product of the enzyme glucose oxidase activity while degrading glucose (Molan and Betts, 2004; Brudzynski, 2006)], acidic pH levels (Gethin et al., 2008), presence of methylglyoxal (MGO) [reacting with macromolecules such as DNA, RNA, and proteins (Adams et al., 2008; Majtan et al., 2014)], among others.

In this work, the antibacterial effect of two lytic bacteriophages vB_EcoS_CEB_EC3a and vB_PaeP_PAO1-D were evaluated either alone or combined with a Portuguese honey, C1, in 24 h biofilms formed in porcine skin explants.

## Materials and methods

### Bacterial strains and growth conditions

Two *Escherichia coli* strains were used in this study: the clinical isolate EC3a that was kindly provided by the Hospital Escala Braga (Portugal) for phage vB_EcoS_CEB_EC3a (EC3a) propagation and the *E. coli* reference strain CECT 434 (purchased from the Spanish Type Culture Collection) for biofilm experiments.

*Pseudomonas aeruginosa* reference strain PA01 (DSM22644), purchased from the German Collection of Microorganisms and Cell Cultures, was used for isolation and propagation of phage vB_PaeP_PAO1-D (PAO1-D) and for biofilm experiments. Other 36 strains of *P. aeruginosa*, were used for PAO1-D host range evaluation that included 3 culture collection strains—ATCC 10145, CECT 111, PAO1—and 33 clinical isolates [Hospital Escala Braga (Portugal)].

Bacteria were cultured at 37°C for ~18 h in Tryptic Soy Broth (TSB, VWR) or Tryptic Soy Agar medium (TSA; TSB containing 1.2% (w/v) agar, NZYTech). MacConckey Agar (Merck®) and Pseudomonas isolation agar (PIA, Sigma-Aldrich) with 5% (w/v) glycerol (Sigma-Aldrich), were used as selective media for *E. coli* and *P. aeruginosa*, respectively, for viable cell counts.

### C1 honey origin, minimum inhibitory concentration and physicochemical characterization

The honey C1 is a single-flower honey from chestnut (92% *Castanea sativa*) collected from the Minho region in Portugal that has a conductivity of 1534 μS.cm^−1^. The minimum inhibitory concentration (MIC) values for *E. coli* and *P. aeruginosa* were determined as described in the guidelines of the Clinical and Laboratory Standards Institute (Andrews and Andrews, 2001; Ferraro et al., 2003) using a honey concentration range from 50% (w/v) to 3,125% (w/v). C1 was physicochemical characterized as previously described (Nishio et al., 2016): the pH was performed as described by the International Honey Commission (Bogdanov, 2002), the color was determined according to the standards already established by the United States Department of Agriculture (USDA) (United States Deparment of Agriculture, 1985), the MGO concentration was obtained by RP-HPLC as described previously (Adams et al., 2008), the protein content was determined using the BCA Protein Assay Kit (Thermo Scientific™ Pierce™) according to manufacturer instructions, and the Hydroxymethylfurfural (HMF) content was determined by White's method (White, 1979).

### Bacteriophage origin and production

The phages used in this work were EC3a for *E. coli*, isolated from raw sewage (Nishio et al., 2016), and PAO1-D, for *P. aeruginosa*, isolated from the Sextaphage commercial cocktail (Microgen, ImBio Nizhny Novgorod, Russia). Each phage was produced in the respective isolation host: EC3a in EC3a strain and PAO1-D in PAO1 strain, using the plate lysis and elution method (Sambrook and Russell, 2001). Briefly, 5 μL of phage suspension were spread evenly on host bacterial lawns using a paper strip and incubated overnight (O/N) at 37°C. Then, 3 mL of SM Buffer (5.8 g.L^−1^ NaCl, 2 132 g.L^−1^ MgSO_4_.7H_2_O, 50 mL.L^−1^ 1 M Tris-HCl pH 7.5, VWR) were added to each plate and re-incubated O/N at 4°C with gentle stirring (50 rpm on a PSU-10i Orbital Shaker 134 (BIOSAN)). The floating liquid was collected and centrifuged (10 min, 9,000 × g, 4°C), and afterwards, phages were concentrated by incubating the lysate with 58.4 g.L^−1^ NaCl for 1 h at 4°C under slow agitation, and the resultant supernatant with 100 g.L^−1^ PEG 8000 (ThermoFisher Scientific) at 37°C O/N. The subsequent suspension was centrifuged, purified with 1:4 (v/v) chloroform, filter sterilized (PES, GE Healthcare, 0.2 μm) and stored at 4 °C until use.

### Phage growth parameters

One-step growth curves of the two phages in the two different strains were performed as described previously (Sillankorva et al., 2008). Briefly, 10 mL of a mid-exponential-phase culture was harvested by centrifugation (7,000 × *g*, 5 min, 4°C) and resuspended in 5 mL fresh TSB medium in order to obtain an OD_600_ of 1.0. To this suspension, 5 mL of phage solution were added in order to have a MOI of 0.001 and phages were allowed to adsorb for 5 min at room temperature. The mixture was than centrifuged as described above and the pellet was resuspended in 10 mL of fresh TSB medium. Samples were taken every 5 min over a period of 1 h and immediately plated.

### Transmission electron microscopy analysis

Phage PAO1-D particles were sedimented by centrifugation (25,000 × g, 60 min, 4°C) and washed twice in tap water by repeating the centrifugation step. Subsequently, the suspension was deposited on copper grids with carbon-coated Formvar films, stained with 2% (w/v) uranyl acetate (pH 4.0) (Agar Scientific), and examined using a Jeol JEM 1400 (Tokyo, Japan) transmission electron microscope (TEM). Images were digitally recorded using a CCD digital camera Orious 1,100 W, Tokyo, Japan.

### Assessment of phage viability in C1 honey

Phage PAO1-D and EC3a viability was tested in C1 honey. For that, 2 × 10^9^ PFU.mL^−1^ were incubated at 37°C with 25% (w/v) and 50% (w/v) C1 honey, mentioned hereafter as C1_25%_ and C1_50%_, respectively. Samples were taken every hour until 6 h, and then after 24 h.

Controls were performed in sterile deionized water instead of honey. For each time point, phages were serial-diluted and quantified by mixing 100 μL of diluted solution with 100 μL of host bacteria culture and with 3 mL of TSA top agar (TSB supplemented with 0.6% (w/v) agar). The mixture was poured onto a layer of TSA (Adams, 1959). After an O/N incubation at 37°C, the plaque forming units (PFU) were determined. Three independent experiments were performed.

### *In vitro* biofilm formation and treatment

The turbidimetry (620 nm) of a 16 h-grown EC3a or PAO1 inoculum was adjusted to 0.13 (corresponding between 2-3 × 10^−8^ CFU.mL^−1^), and 10-fold diluted in TSB in order to have an initial inoculum concentration of 10^−7^ CFU.mL^−1^ (Crouzet et al., 2014; Pires et al., 2017). For biofilm formation, 200 μL of the bacterial suspension were added to wells of a 96-well plate that was subsequently incubated for 24 h at 37°C and 120 rpm [orbital shaker ES-20/60 214 (BIOSAN)]. For the formation of dual-species biofilms, the turbidimetry of both bacteria was adjusted to 0.13 and 5-fold diluted in TSB. After, 100 μL of each suspension were added to the wells and biofilm formation allowed to proceed as described above.

Phage treatments were performed with 1 × 10^9^ PFU.mL^−1^ and honey challenge was done with C1_25%_ and C1_50%_. Monospecies biofilms formed during 24 h, were washed twice with saline [0.9% (w/v) NaCl, VWR] to remove non-adhered cells. After, 200 μL of phage, honey or 100 μL of phage 2 × concentrated and 100 μL of honey 2 × concentrated were added to each well and plates were incubated at 37°C, 120 rpm [orbital shaker ES-20/60 (BIOSAN)]. Dual-species biofilms were treated with 100 μL of the *P. aeruginosa* phage and 100 μL of *E. coli* phage, 200 μL of honey or with 50 μL of the *P. aeruginosa* phage and 50 μL of the *E. coli* phage both 4 × concentrated and 100 μL of honey 2 × concentrated. The control samples were performed with 100 μL of 2 × TSB, and 100 μL of SM buffer. Samples were analyzed at 0, 6, 12, and 24 h for viable cell quantification. At each sampling time point, biofilms were washed twice with saline [0.9% (w/v) NaCl, VWR], 200 μL saline added to each well and all biomass detached from the polystyrene bottom and side wall, by scraping, before CFU analysis. Three independent experiments were performed in triplicate.

### Preparation of porcine skin explants

Fresh porcine skin explants were generously supplied by ICVS - Life and Health Sciences Research Institute (Braga, Portugal), immediately stored in vacuum at −20 °C and thawed only before use.

Explants were cut into 2 × 2 cm pieces and disinfected as described previously (da Costa et al., 2015). After disinfection, each skin piece was placed between two sterile stainless steel plates with an o-ring in the center, to delimit the infection region. The skin was immobilized by fixing the upper metal plate with wing nuts (da Costa et al., 2015).

### *Ex vivo* biofilm formation and treatment

Three different biofilm treatments were evaluated: phage, honey and the combination of both agents. Similar to *in vitro* treatments, 1 × 10^9^ PFU.mL^−1^ of phages EC3a and PAO1-D, and C1_25%_ and C1_50%_ were used. The combinatorial effect of phage-honey was accomplished using the concentrations used in the single-agent experiments.

For monospecies biofilm formation, 80 μL of the bacterial suspension prepared as described above were placed in direct contact with the skin inside of the O-ring, and for dual-species biofilms 40 μL of both bacterial suspensions were used. The stainless steel plates holding the skins were placed in previously disinfected desiccators and incubated for 24 h at 37°C.

The infected area was washed twice with saline and after, in monospecies biofilms 80 μL of phage, honey or both agents were placed in the O-ring area, and incubated at 37°C. In dual-species biofilms the volumes of each agent were: 40 μL of each phage 2 × concentrated; 80 μL of honey; or 20 μL of each phage 4 × concentrated and honey 2 × concentrated. Biofilm cells were collected with the aid of a cotton swab that was then immersed in 1 mL saline. The suspension was centrifuged (8,000 × g, 10 min, 4°C) and the pellet resuspended in 1 mL saline. Samples were analyzed at 0, 6, 12 and 24 h, for viable cell quantification. Three independent experiments were performed in triplicate.

### Quantification of viable cells from biofilms

Viable cells in biofilms were quantified by adapting a previously described method (Pires et al., 2017). Serial dilutions were performed in saline containing 1 mM ferrous ammonium sulfate (FAS, Applichem Panreac) to assure that all non-infecting phages were destroyed (Park et al., 2003). Samples (10 μL) were plated on MacConkey Agar or PIA plates, for *E. coli* or *P. aeruginosa* cell counts, respectively, using the microdrop technique (Naghili et al., 2013). Plates were incubated 16 h at 37°C, and colony forming units (CFU) were determined.

### Interpretation of the results

For each combined therapy (phage EC3a, phage PAO1-D, honey C1_25%_, honey C1_50%_) we determined if the outcome of the combination was synergistic according to the methodology described by Chaudhry et al. (2017). In brief, an outcome was regarded as synergistic when the equation Log(C)-log(S_P_)-log(S_H_)+log(S_PH_) < 0 was valid. In the equation, C refers to the cell density obtained in the control (no treatment), and S_P_, S_H_, and S_PH_ are the surviving cell densities after treatment with phage (P), honey (H), or both combined (PH), respectively (Chaudhry et al., 2017). The calculations are presented only for monospecies biofilms formed on porcine skin, and dual-species biofilms formed on polystyrene and porcine skin (Table S3).

### Statistical analysis

Statistical analysis of the results was performed using GraphPad Prism 6. Mean and standard deviations (SD) were determined for the independent experiments and the results were presented as mean ± SD. Results were compared using Two-way ANOVA, with Tukey's multiple comparison statistical test. Differences were considered statistically different if *p* ≤ 0.05 (95% confidence interval).

## Results

### C1 honey physicochemical characterization and MIC determination

C1 honey is a white honey with a pH of 5.4. The total protein content is 81.7 mg.kg^−1^, the MGO concentration 1000.2 mg.kg^−1^ and the HMF is < 4 mg.kg^−1^. The MIC experiments of C1 on *E. coli* and *P. aeruginosa* was 12.5% (w/v) and 25% (w/v), respectively.

### Phage growth parameters and morphology

Phage EC3a is a strictly virulent *Siphovirus* that has already been partly characterized (Nishio et al., 2016). EC3a has a latent period of ~15 min giving rise to ~53 progeny per infected cell.

**Figure 1:**
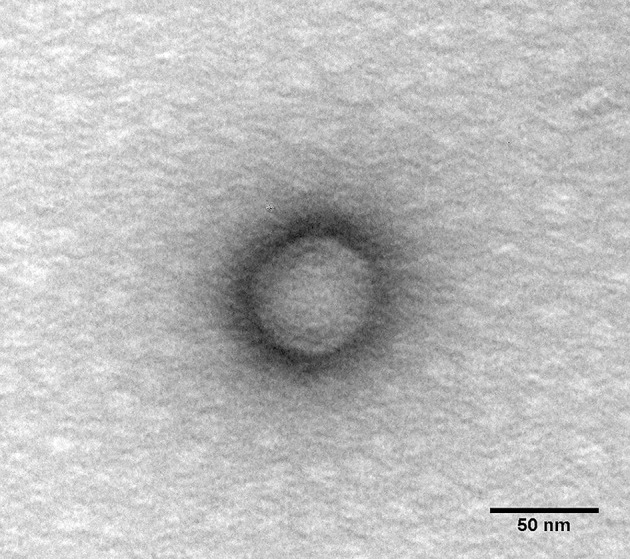
TEM micrograph of PAO1-D bacteriophage particle (scale bar 50 nm).

**Figure 2:**
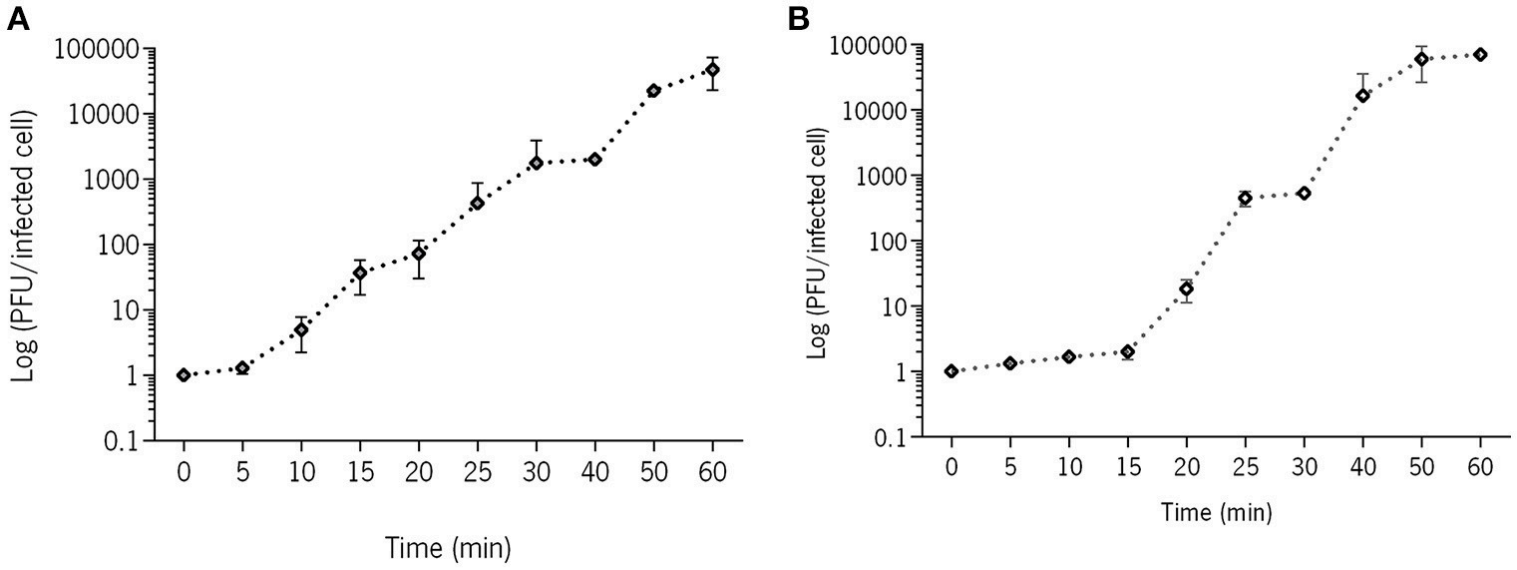
One-step growth curve of phages **(A)** PAO1-D and **(B)** EC3a in their respective hosts. Error bars represent standard deviations from 2 independent experiments performed in duplicate.

PAO1-D, isolated from the Sextaphage preparation, is a *Podovirus* showing a 56 nm × 64 nm icosahedral capsid, and a 12 nm non-contractile tail (Figure 1). This phage was selected for all further experiments based on its lytic spectra toward the clinical isolates (Table S1) and also on the dimension of its large halo (Table S2, Figure S1) that may suggest the presence of enzymes with higher efficiency to degrade the EPS matrix of biofilms. PAO1-D has a short latent period (5 min) and a burst size of ~61 phages per infected cell (Figure 2).

### Phage viability in C1 honey

**Figure 3:**
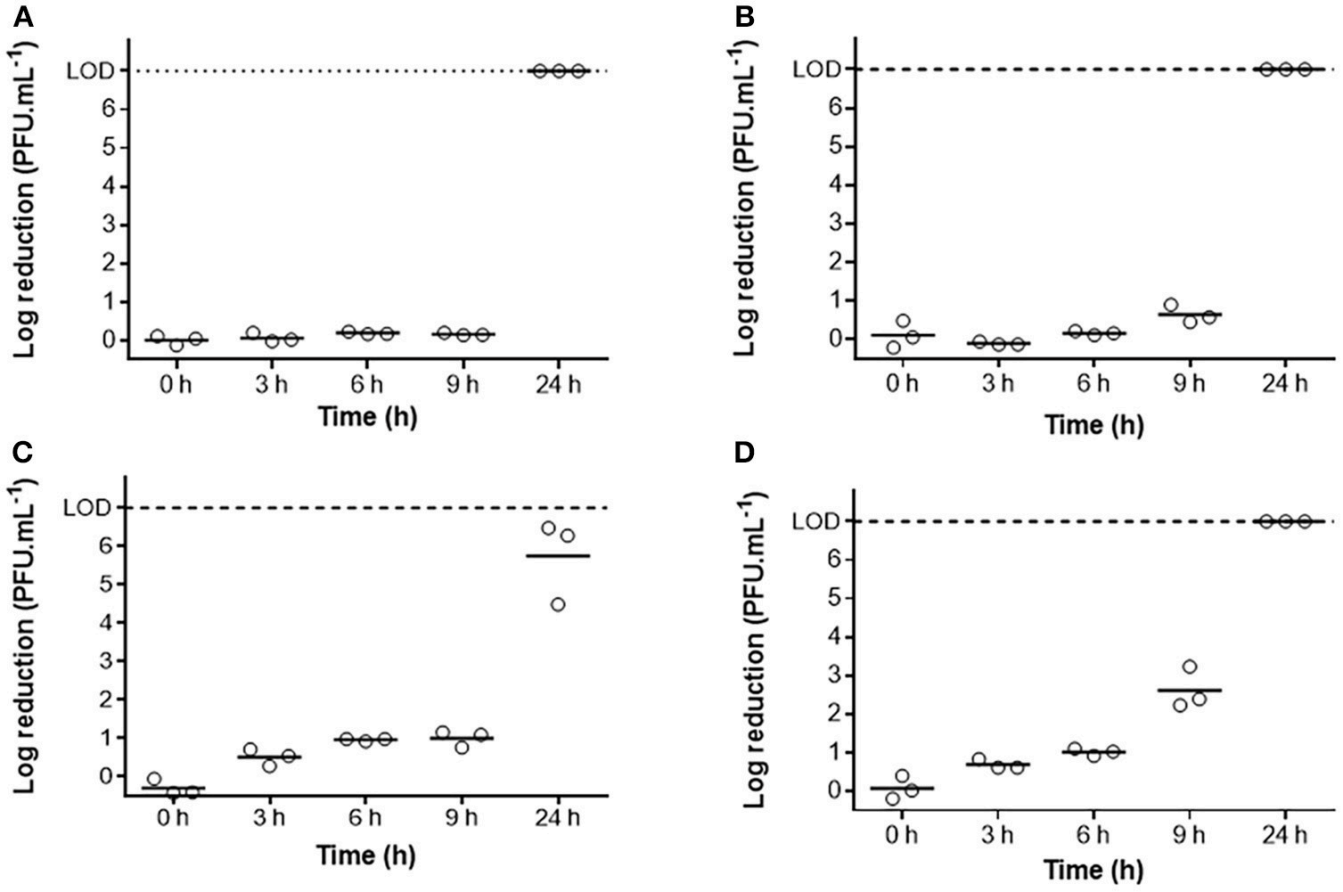
Viability (PFU.mL^−1^) of phage EC3A and PAO1-D over 24 h in C1 honey: **(A)** EC3a in C1 25%, **(B)** EC3a in C1 50%, **(C)** PAO1-D in C1 25%, and **(D)** PAO1-D in C1 50%. Each independent assay (o) and the mean (–) are represented. LOD (Limit of Detection) = 7-Log.

Viability of both phages, EC3a and PAO1-D, was assessed on C1_25%_ and C1_50%_ (Figure 3). Until 9 h, there was no evident effect on EC3a viability in C1_25%_ and C1_50%_. However, no infective EC3a were recorded after 24 h of contact with both concentrations of C1 (*p* < 0.05). Furthermore, although PAO1-D showed to be slightly more sensible to honey until 9 h of contact, C1_25%_ did not cause complete inactivation of this phage at 24 h.

### *E. coli* and *P. aeruginosa* colonization of surfaces

**Figure 4:**
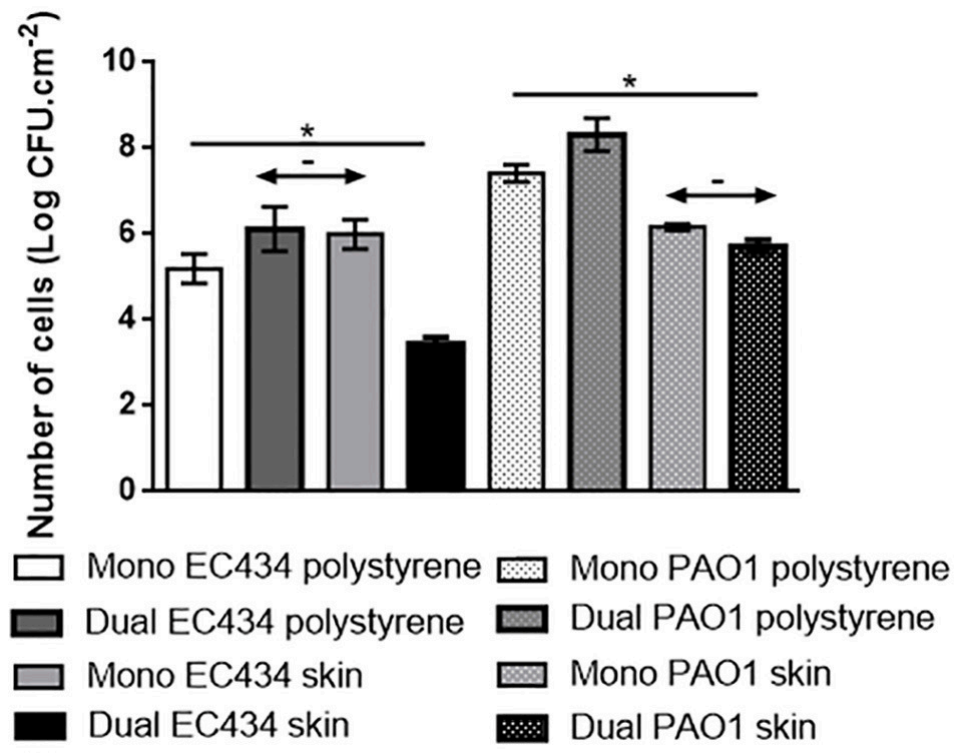
Bacterial colonization (CFU.cm^−2^) of 24 h-old biofilms of *E. coli* 434 and *P. aeruginosa* PAO1 formed individually (Mono) or combined (Dual). Number of cells in dual species biofilms were counted separately on MacConkey for *E. coli* or PIA for *P. aeruginosa*. Data are shown as mean ± SD and results. With the exception of the two arrowheads with “-” all other values were considered statistically different (*p ≤ 0.05).

*E. coli* CECT434 and *P. aeruginosa* PAO1 colonization was assessed in 24 h mono- and dual-species biofilms formed *in vitro* and in porcine skin explants (Figure 4). Although, monospecies biofilms of *E. coli* colonized better the skin surfaces than polystyrene, no significant differences in colonization were observed for monospecies *P. aeruginosa* biofilms (*p* < 0.05). Dual-species biofilms of both bacteria on polystyrene presented statistically more cells than monospecies biofilms formed in this material. The colonization of porcine skins by *E. coli* and *P. aeruginosa* alone and when mixed was also analyzed. In general, the level of colonization by *P. aeruginosa* was similar in both experiments, however the colonization by *E. coli* was highly influenced by the presence of *P. aeruginosa* resulting in less 2.6-Log cells than in monospecies *E. coli* biofilms (*p* < 0.05).

### Antibiofilm effect of honey and phage on polystyrene–formed biofilms

**Figure 5:**
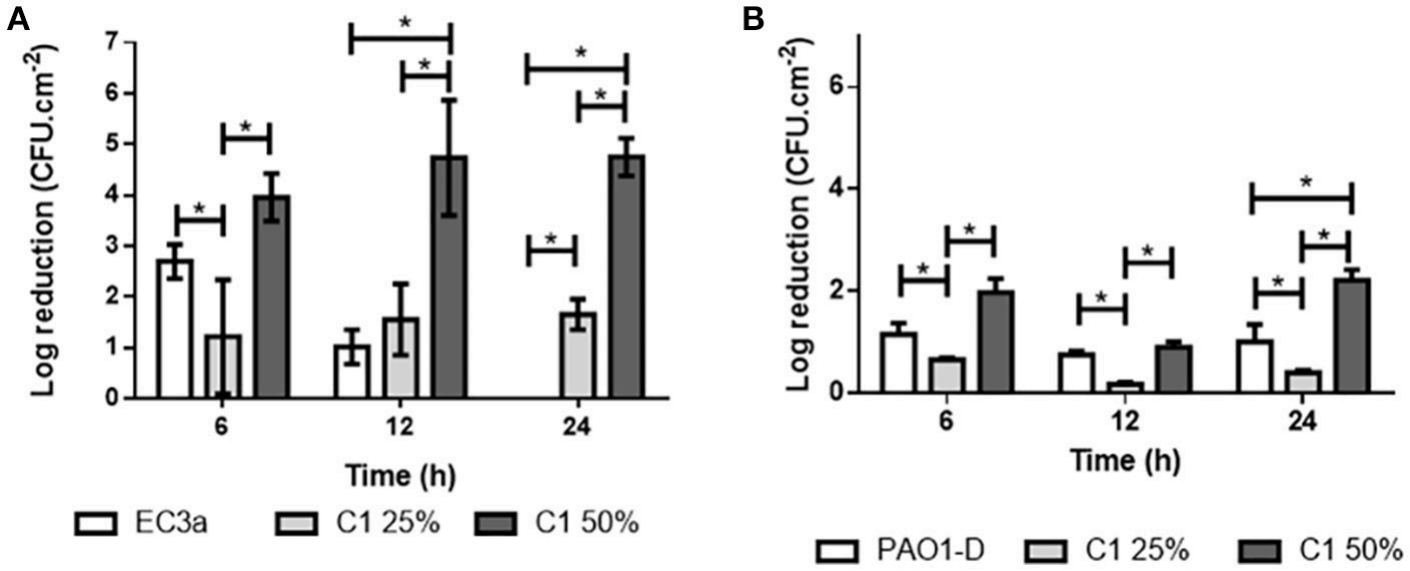
Antibiofilm effect of phage and honey against monospecies biofilms formed in polystyrene surfaces: **(A)** EC3a, C1_25%_ and C1_50%_ on *E. coli* biofilms and **(B)** PAO-D, C1_25%_ and C1_50%_ in *P. aeruginosa* biofilms. Data are shown as mean ± SD and results were considered statistically different if *p ≤ 0.05.

The effect of phage and honey was evaluated in 24 h-old biofilms formed in 96-well polystyrene plates (Figure 5).

Phages EC3a and PAO1-D were used against *E. coli* and *P. aeruginosa* biofilms, respectively. EC3a antibiofilm activity was highest after 6 h of infection, reducing about 2.7-Log *E. coli* viable cells. However, no effect of EC3a was noticed after 24 h. Contrarily to EC3a, phage PAO1-D caused a uniform cell reduction throughout the 24 h experiment that was always higher compared to the reductions caused by C1_25%_.

The effect of C1_25%_ on cell count reductions was always less evident (varied form a 1.2 to a 1.6-Log reduction) than the effect of C1_50%_ that varied from 4.0-Log to a 4.7-Log reduction in the time-points assessed.

C1_50%_ showed always superior antibiofilm activity compared to the lower honey concentration and also significantly higher killing capacity than both tested phages at 24 h of treatment (*p* < 0.05).

### Antibiofilm effect of phage, honey, and phage-honey combination on dual-species biofilms formed on polystyrene

**Figure 6:**
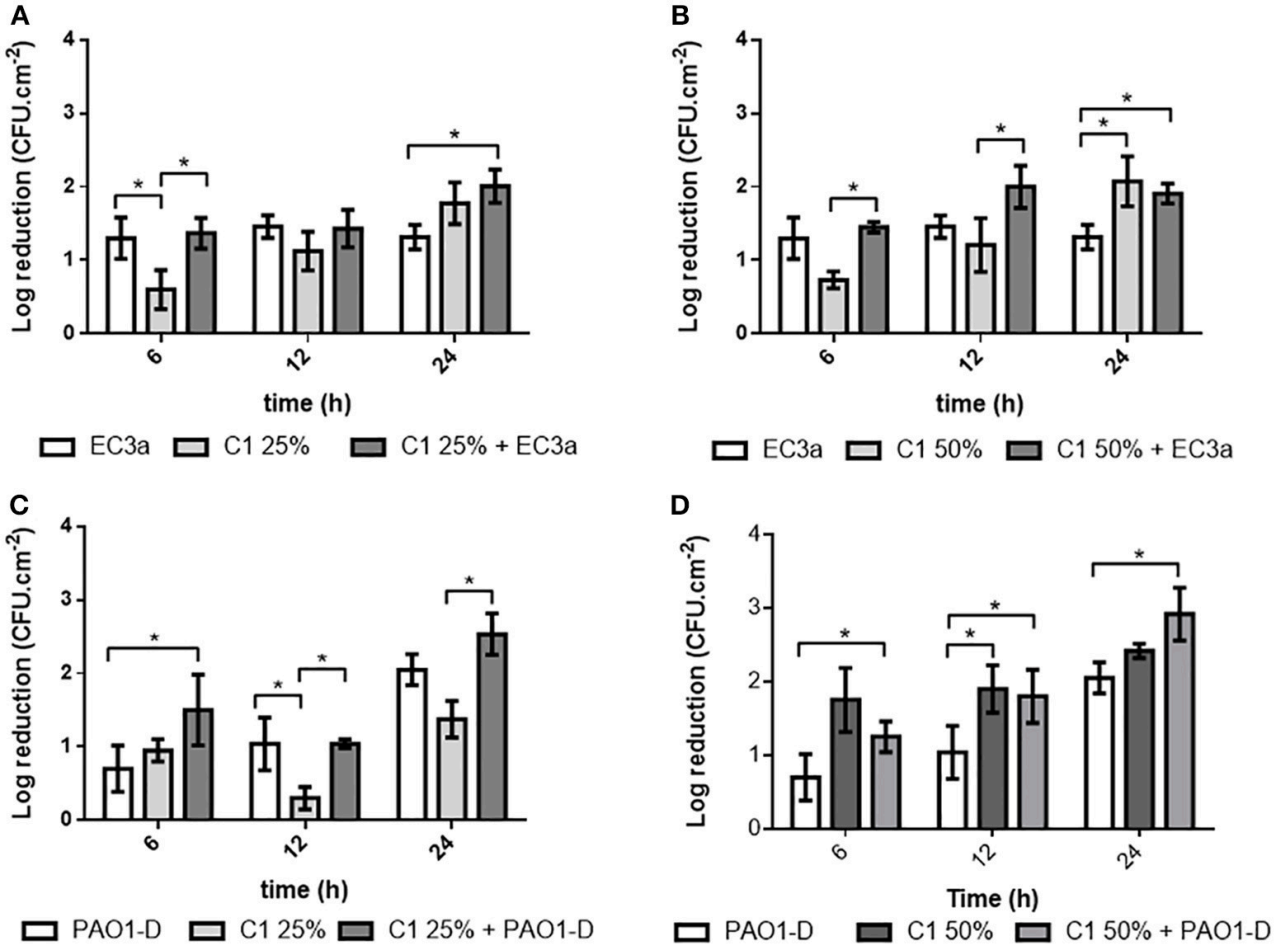
Antibiofilm effect of phage, honey and combined therapy against dual-species biofilms formed in polystyrene surfaces: **(A)** EC3a and C1_25%_ on *E. coli*; **(B)** EC3a and C1_50%_ on *E. coli*; **(C)** PAO1-D and C1_25%_ on *P. aeruginosa*; **(D)** PAO1-D and C1_50%_ on *P. aeruginosa*. Data are shown as mean ± SD and results were considered statistically different if *p ≤ 0.05.

Dual-species biofilms formed on polystyrene were challenged with a cocktail of both phages, honey or all combined (Figure 6). *E. coli* cell reductions observed at 6 h of combined honey-phage treatment were in great part due to phage EC3a resulting in similar values to those obtained when applying phage alone. Honey alone resulted in a gradual increase of the number of *E. coli* cells killed from less than 1-Log at 6 h to 1.8–1.9-Log at 24 h with C1_25%_ and C1_50%_, respectively. At 12 h, honey at 50% combined with phage was significantly better (*p* < 0.05) than honey alone. By 24 h of treatment, the decrease of cells obtained when a combined therapy was used was, contrarily to the initial 6 h time point, greatly due to the action of C1 honey. Nonetheless, honey alone was never as efficient in killing *E. coli* living in dual-species biofilms compared to its effect on monospecies *E. coli* biofilms (compare Figures 5, 6).

In terms of treatment effects on *P. aeruginosa* present in the dual-species biofilms, overall the numbers of cells killed gradually increased with phage and both honey concentrations alone. The combined treatment using phage and honey C1_25%_ was statistically higher (*p* < 0.05) than the action of phage alone at 6 h than honey alone at 12 and 24 h (*p* < 0.05). Phage combination with honey C1_50%_ was not significantly different from the action exerted by honey alone (*p* > 0.05).

Even though statistically significant differences where observed after the different combinations, overall the antibacterial action of EC3a phage until 12 h was better than honey, at both concentrations, in killing *E. coli* from dual-species biofilms. Phage PAO1-D had a similar or slightly better effect than honey at 25%. Combined treatment resulted in a slightly better antibacterial outcome than phage and honey alone however without resulting in a synergy effect (see Table S3).

### Antibiofilm effect of phage, honey, and the phage-honey combination on 24 h-old porcine skin-formed biofilms

**Figure 7:**
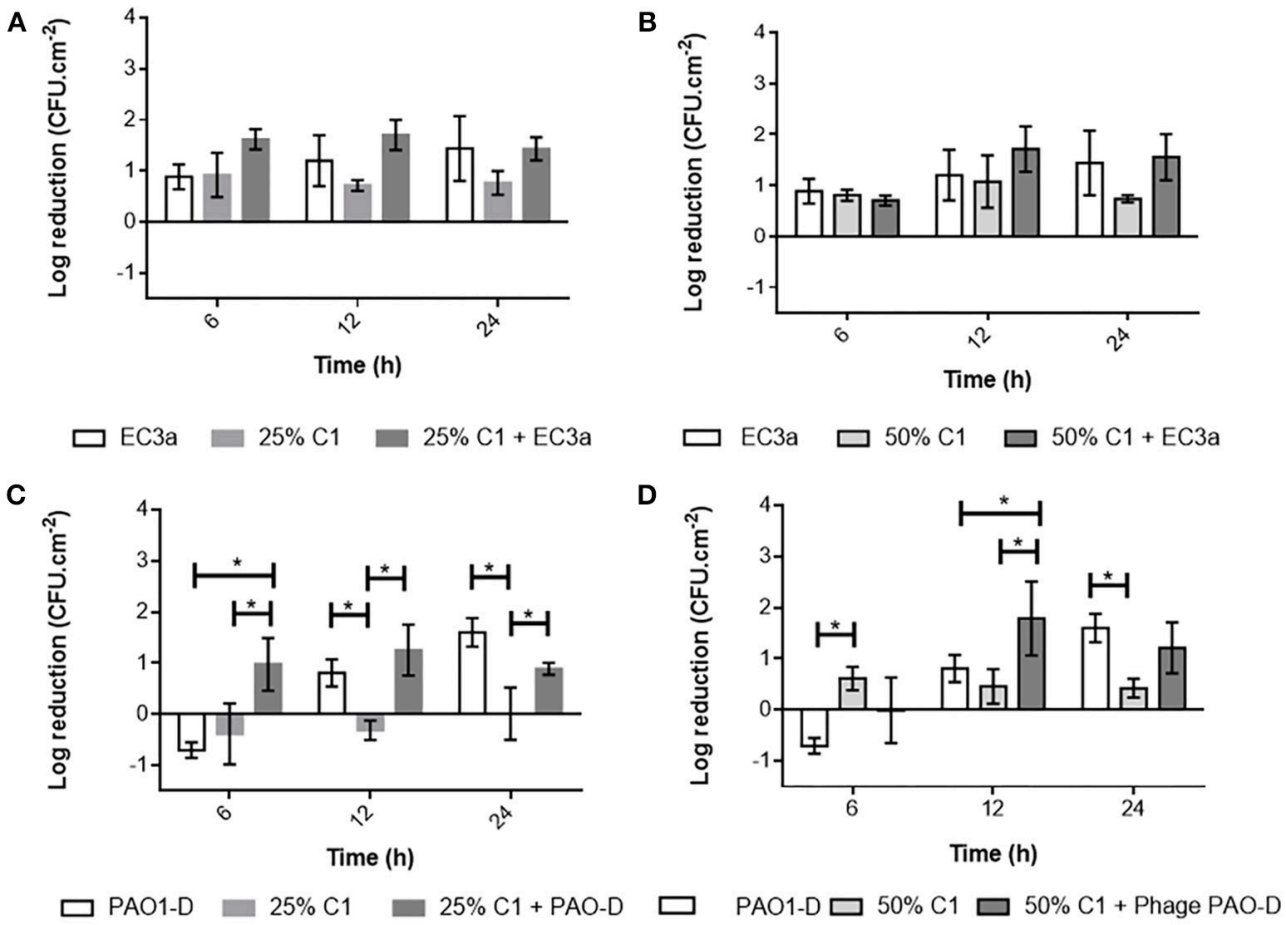
Antibiofilm effect of phage and honey against monospecies biofilms formed in pig skin: **(A)** EC3a and C1_25%_ on *E. coli*; **(B)** EC3a and C1_50%_ on *E. coli*; **(C)** PAO1-D and C1_25%_ on *P. aeruginosa*; **(D)** PAO1-D and C1_50%_ on *P. aeruginosa*. Data are shown as mean ± SD and results were considered statistically different if *p ≤ 0.05.

The effect of phage, honey and also the combination of both antimicrobial agents was evaluated in *E. coli* and *P. aeruginosa* monospecies biofilms formed in porcine skin explants (Figure 7).

Considering *E. coli* biofilms, the effect of phage EC3a in the *ex vivo* model was constant (*p* > 0.05) (~1-Log reduction in average) from 6 to 24 h (contrarily to the 6 h reduction observed over time in the *in vitro* assay). A similar consistency was observed with the C1_25%_ or C1_50%_ effect in the porcine skin, ~1-Log viable cell reduction over 24 h.

Throughout the experiment, the combination of EC3a and C1_25%_ or C1_50%_ were statistically similar to, at least, one of the antimicrobial agents used separately (*p* > 0.05). The exception was observed 6 h after treatment with EC3a and C1_25%_ when the reduction of viable cells was higher with the combination (1.6-Log) comparatively to phage (0.9- Log) or honey (0.9-Log) (*p* < 0.05).

Regarding *P. aeruginosa* biofilms, C1_25%_ had no effect on cell reduction (there was even an increase in CFU count at 6 and 12 h after treatment), while C1_50%_ reduced cell concentration in no more than 0.6-Log during the 24 h treatment.

The antibiofilm effect of PAO1-D increased over time (*p* < 0.05) leading to 1.6-Log cell reduction after 24 h of treatment.

The combination of PAO1-D with C1_25%_ led to a constant effect along the 24 h experiment (*p* > 0.05) with a maximum viable cell reduction at 12 h (1.3-Log reduction). PAO1-D combined with C1_50%_, also displayed highest reduction of viable cells at 12 h (1.8-Log cell reduction).

### Antibiofilm effect of phage, honey, and phage-honey combination on dual species biofilms formed in porcine skin

**Figure 8:**
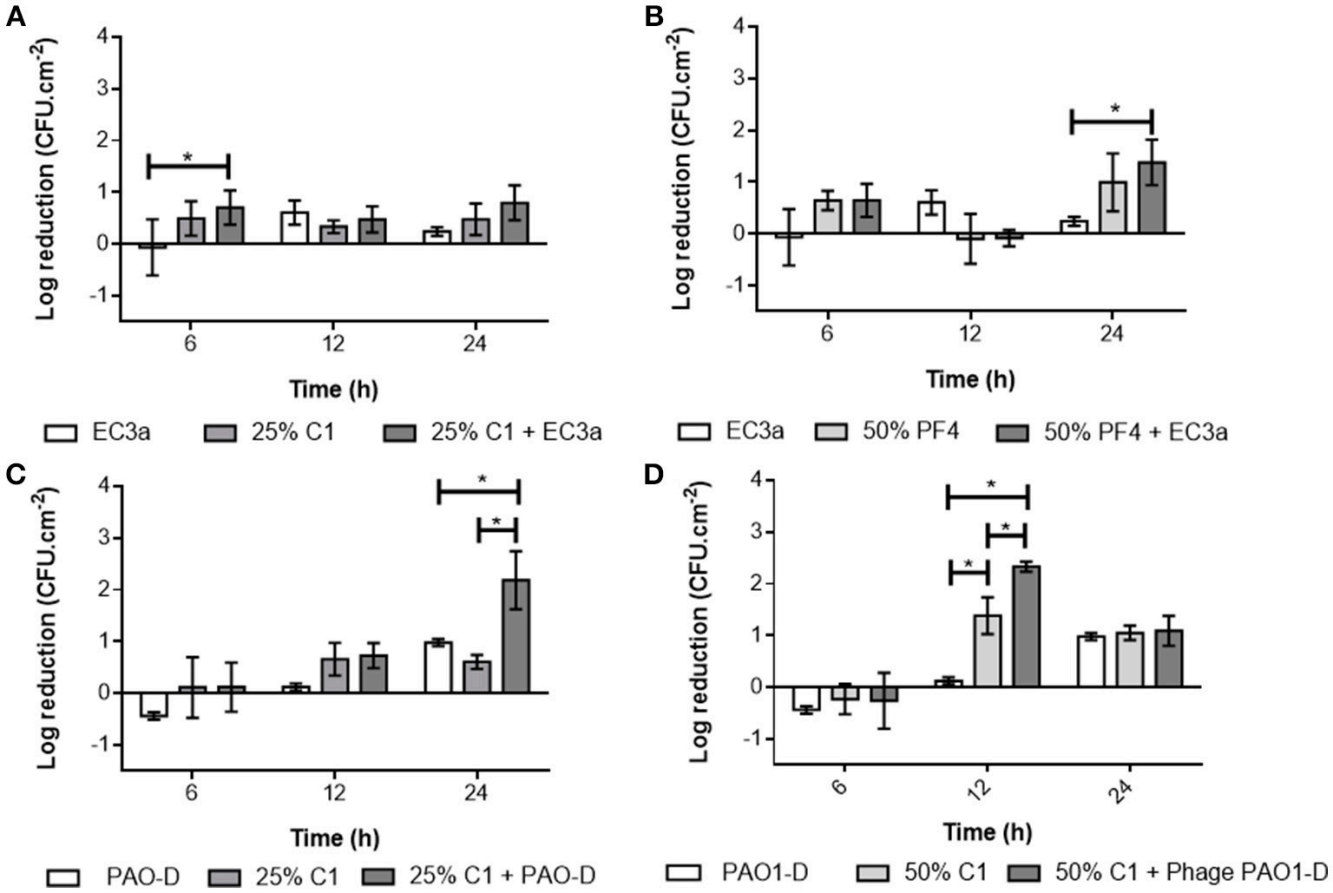
Antibiofilm effect of phage, honey, and honey-phage combinations against dual-species biofilms formed in pig skin: **(A)** EC3a and C1_25%_ on *E. coli*; **(B)** EC3a and C1_50%_ on *E. coli*; **(C)** PAO1-D and C1_25%_ on *P. aeruginosa*; **(D)** PAO1-D and C1_50%_ on *P. aeruginosa*. Data are shown as mean ± SD and results were considered statistically different if *p ≤ 0.05.

The antibiofilm effect of phage, honey, and their combination was tested against dual-species biofilms of *E. coli* and *P. aeruginosa* formed on porcine skin and the effect reported per bacterial species, respectively (Figure 8).

The antibiofilm effect of EC3a against *E. coli* in dual-species biofilms was similar among time points showing a 0.6-Log reduction, in average, at 12 h post-infection. Similarly, C1_25%_ alone maintained a cell reduction below 0.5-Log in all analyzed samples, and C1_50%_ alone below 1.0-Log. The combination of phage EC3a with C1_25%_ didn't vary considerably throughout time resulting in ~0.5 to 0.8-Log reductions of *E. coli* viable cells from dual-species biofilms, while the combination of EC3a + C1_50%_ varied from no cell reductions up to 1.4-Log reduction, at 24 h post-treatment, of *E. coli* from dual-species biofilms.

Concerning *P. aeruginosa* reductions in dual-species biofilms, C1_25%_ alone contributed with no more than 0.9-Log observed at 12 h, while C1_50%_ displayed a 1.8-Log reduction in the same period. On the other hand, the effect of PAO1-D on *P. aeruginosa* increased significantly from 6 to 24 h (*p* ≤ 0.05).

The combination of PAO1-D and C1_25%_ revealed a synergistic effect 24 h after treatment (Figure 8 and Table S3), causing an average cell reduction of 2.2-Log, higher than the sum of phage and honey alone [1.0-Log (PAO1-D) + 0.6-Log (C1_25%_)]. Synergism was also observed for C1_50%_ combined with phage, at 12 h: 2.3-Log > 0.1-Log (PAO1-D) + 1.4-Log (C1_50%_) (see also result in Table S3).

Overall, honey and phage were more effective in controlling *P. aeruginosa* in dual-species biofilms formed in porcine skin than in monospecies *P. aeruginosa* biofilms.

## Discussion

The reduction of bacterial wound bioburden to host-manageable levels, as well as the elimination of certain virulent forms of wound pathogens has become a goal of the wound care professionals. In fact, a direct link between bacterial load and subsequent healing has been demonstrated (Bendy et al., 1964) being the successful closure of wounds apparently dependent on maintaining a bacterial level below 10^5^ CFU.g^−1^ of tissue (Robson, 1997; Bowler, 2003). In the wound-healing scheme, the use of alternative antimicrobial agents is considered when other approaches as the use of moisture-retentive dressings (that assist the hosts' phagocytic defense mechanisms by creating a moist wound environment) have been unsuccessful. These alternative antimicrobial agents are expected to supplement the host immune activity in reducing wound bioburden until a balance in favor of the host is restored. The antimicrobial potential of honey and phage to control biofilm-related infections can be a potentially good alternative for topical applications, particularly for treatment of chronic wounds. Phage therapy effectiveness (orally and locally administered) in chronic suppurative infections of the skin caused by *Pseudomonas, Staphylococcus, Klebsiella, Proteus* and *Escherichia* was described over 30 years ago with ~50% of the 31 studied patients resulting in an “outstanding” improvement (Cislo et al., 1987). In 2002, a phage impregnated polymer used to treat infected venous stasis skin ulcers achieved complete healing in 70% of the 107 patients (Markoishvili et al., 2002). Research using animal models has also supporting evidences of phage safety and efficacy in treating chronic wounds infected by *S. aureus, P. aeruginosa* and *Acinetobacter baumannii* (Mendes et al., 2013; Seth et al., 2013). Recent works also have shown superior chronic wound healing rates and a lower healing time of honey when compared to commonly used products (Sharp, 2009; Imran et al., 2015). For example, Medihoney dressing used in non-healing venous leg ulcers during 12 weeks revealed a decrease in ulcer pain, size and odor (Dunford and Hanano, 2004). Bacteriological changes in venous leg ulcers treated with Manuka honey or hydrogel were evaluated in 108 patients (Gethin and Cowman, 2008), and after 4 weeks, Manuka honey was able to eradicate MRSA in 70% of treated wounds.

Chronic wounds are usually polymicrobial in nature and therefore, this work focused on evaluating the interaction of honey and phages, alone and both combined, with dual-species biofilms of *P. aeruginosa* and *E. coli*.

One essential step before carrying the combined antimicrobial therapy was to assess the viability of phages in honey. Although our phage collection comprises some fully characterized *P. aeruginosa* phages (Pires et al., 2011, 2014, 2015, 2017), all showed to be highly sensitive to this chestnut honey. Therefore, isolation of phages from the Sextaphage and also from Intestiphage preparations (both from Microgen, Russia) using PAO1 as host strain allowed the isolation of phages presenting an increased insensitivity to the chestnut honey used. Different phages were isolated, however PAO1-D showed the best features (Table S1, Figure S1) and was therefore chosen for the antimicrobial experiments. The *E. coli* phage on the other hand was chosen taking advantage of its known morphologic and genomic characteristics, and also due to its known survival on two polyflora honeys (Oliveira et al., 2017) during the first 6 h. In this work, chestnut honey partially or completely destroyed both phages after 24 h of exposure resulting in phage concentrations below the limit of detection. The main characteristics of honey that seem to cause loss in phage viability are its low pH (between 3.2 and 4.5), high sugar content (about 80%) that can cause an osmotic shock, possible presence of proteases, and the slow release of hydrogen peroxide when dissolved in water (about 1 mmol.L^−1^) (Rossano et al., 2012; Agún et al., 2018). Nevertheless, this late destruction of phage particles, only at 24 h, grants them capacity to complete several infection cycles before destruction since both phages have relatively short latent and burst periods (Figure 2).

The antimicrobial actions of each of these agents have distinct mechanisms in biofilms. While phages specifically destroy bacteria through host-receptor recognition and infection, honey reaches the same destruction by oxidative stress, osmotic pressure, acidity, hydrogen peroxide release, presence of methylglyoxal (MGO) among other mechanisms. We recently observed an enhanced antibacterial effect of phage and other two Portuguese honeys in monospecies biofilms of *E. coli* formed *in vitro*, and these results led us to pursue research with other honey types, and other bacteria, this time using a more complex and already validated model—the porcine skin explant model (da Costa et al., 2015). The results obtained where nonetheless compared with those obtained using the easiest and most commonly used high-throughput biofilm model—the polystyrene microplate biofilm model.

Analyzing the effect of the phages alone, the two lytic phages tested, EC3a and PAO1-D, decreased *E. coli* and *P. aeruginosa* cells from biofilms formed in polystyrene and porcine skin, respectively. The phages action against biofilms formed in porcine skin explants increased with time when compared with *in vitro*-formed biofilms (compare Figures 5, 7). Furthermore, between 6 and 24 h no bacterial regrowth on porcine skins experiments challenged with phage was observed, suggesting a reduced emergence of phage-resistant phenotypes. This phenomenon might be due to the lower cell reductions achieved by EC3a in the later model (1.9-Log) compared to the reductions observed in the polystyrene experiments (2.7-Log) which minimizes the adaptation of *E. coli* to evade EC3a infection.

The honey used in the experiments is a monofloral honey (92% *Castanea sativa*). Regarding the feasibility of safeguarding the inter-lot reproducibility of a honey-based product, the use of honey with a single floral source, as happens with manuka honey (at least 70% of its pollen content should come from *Leptospermum scoparium*) seems to be more convenient. Besides, the chestnut honey has already been reported to have high antimicrobial effect against *E. coli* (Coniglio et al., 2013) and, together with Manuka honey, against *P. aeruginosa* including PAO1 (Hao et al., 2012; Voncina et al., 2015; Bolognese et al., 2016). The tissue of chestnut plants contains compounds such as tannins and antioxidants (Hao et al., 2012), which have inhibitory effects on microorganisms, and 3-aminoacetophenone is the main volatile compound occurring specially in this floral source, known as having antibacterial properties (Bonaga and Giumanini, 1986). Contrarily to phages, the antimicrobial effect of chestnut honey was evident *in vitro*, when the polystyrene-formed monospecies biofilms were treated with a 50% (w/v) honey preparation resulting in a maximum of 5.6-Log and 2.8-Log reductions from *E. coli* and *P. aeruginosa* biofilms, respectively. This is in accordance with the obtained MIC results showing that a lower concentration of C1 honey is needed in order to eradicate *E. coli* in the suspension form, compared with *P. aeruginosa*. However, in an *ex vivo* context, honey was less effective toward *P. aeruginosa*. The lower sensitivity of *P. aeruginosa* to honey might be due to the lower permeability of its cell wall to antimicrobial compounds and its ability to grow in an environment with higher MGO levels. A study led by Kilty in 2011 tested the effect of different MGO concentrations on different strains of *P. aeruginosa* biofilms, within a range of 1800–7300 mg.kg^−1^. According to these tests, MGO concentrations from 3600 to 7300 mg.kg^−1^ were required to reduce the biofilm biomass of different *P. aeruginosa* strains (Kilty et al., 2011). A study led by Lu in 2013 supports this hypothesis, where *P. aeruginosa* was shown to have a higher tolerance to MGO than *Bacillus subtilis, E. coli*, and *S. aureus* (Lu et al., 2013). On the other hand, the active compounds of C1 seemed to be able to diffuse through the EPS matrix of established *E. coli* biofilms reaching and causing damage to the bacterial cells as reported previously (Oliveira et al., 2017). For instance, Lee et al. (2011) demonstrated that even at low concentrations, honey was able to reduce the colonization and subsequent biofilm formation, and virulence of a pathogenic *E. coli* strain assessed by crystal violet staining of the total biofilm biomass, and analysis of expression of quorum sensing and virulence genes. Furthermore, these authors observed that curli fibers, a common factor controlling biofilm formation in *E. coli* 0157:H7, were repressed by acacia honey.

Although honey was not nearly as effective against biofilms formed in porcine skin explants compared to those formed on polystyrene, it can be highlighted that honey provides other properties that may be interesting for wound treatment purposes, such as its role in tissue regeneration (Majtan, 2014; Oryan et al., 2016; Mohamed, 2017).

In this work, we aimed to determine whether the combined treatments with phage and honey exerted an enhanced antibacterial outcome and synergy testing was not the main interest of this study. There is an extensive literature regarding terminology of combined treatment outcomes and how these are described (Greco et al., 1992; Piggott et al., 2015). We use the term “synergy” when the combined phage-honey treatment kills a greater fraction than the effect of the two agents independently and for the interpretation of the results we adopted the approach described by Chaudhry et al. (2017).

The combined treatment using phage and honey against dual-species biofilms formed on polystyrene (Figure 6) caused a slightly improved killing activity compared to the addition of phage or honey alone however, never resulting in a synergistic effect (Table S3). Comparing the efficacy of both phages against mono and dual-species biofilms, while in monospecies biofilms the ability of phages to infect decreased over time, possibly due to the emergence of phage resistant phenotypes, the same was not observed in the presence of another bacterial species where phages continued to be able to reduce cells over the 24 h-period assessed (compare Figures 5, 6).

Monospecies biofilms of *E. coli* and *P. aeruginosa* formed on porcine skins were also targeted using combined therapy. *E. coli* cell numbers started being reduced upon application of EC3a or both honey concentrations and resulted, overall, in slightly more cells killed using the combined therapy approach. On the other hand, *P. aeruginosa* biofilms increased in numbers after 6 h of application of both phage and honey at 25% but surprisingly the combined treatment exerted antibacterial effect. As already described above for the treatment of biofilms formed on polystyrene, honey alone presented lower efficacy against *P. aeruginosa* biofilms formed on porcine skins than *E. coli*.

In dual-species biofilms of *E. coli and P. aeruginosa* only *P. aeruginosa* had a real benefit in dual-species biofilms, clearly outnumbering *E. coli*. This has already been described before and by Cerqueira et al. (2013) whom also analyzed the biofilm structure by confocal laser scanning microscopy and found that these species colonize surfaces forming co-aggregated biofilm organization (Cerqueira et al., 2013). Dual-species biofilms formed on porcine skin explants were not as easily reduced by phage and honey as monospecies biofilms.

However, their removal using the combinatorial phage-honey approach was beneficial at 24 h with C1_25%_ and phage PAO1-D and at 12 h with C1_50%_ also with phage PAO1-D resulting in a synergy outcome. *P. aeruginosa* control on porcine skin explants was more efficient when this bacterium was in the presence of *E. coli*. Moreover, synergistic effects on dual-species biofilm control were observed for both honey concentrations combined with phage although at different time points. These results suggest that in this context honey and phage are enhancing each other's antimicrobial properties. Even though we use the “synergy” terminology, we are aware that this does not give evolutionary dynamics during treatment since these results are limited to the time points assessed (6, 12, 24 h). The validation of the synergy outcome should also be further confirmed employing approved standard methodology for synergy testing (Breitinger, 2012).

Possibly, this is due to honeys penetration through the biofilm EPS as demonstrated with *S. aureus* biofilms (Lu et al., 2014) that will then allow that the phages used can more easily access the target host cells. Taking into account the direct link between bacterial load and subsequent wound healing described above (bacterial levels < 10^5^ CFU.g^−1^ of tissue), in our work we observed a cell load below the 10^5^ CFU.cm^−2^ for *E. coli* 434 and *P. aeruginosa* PAO1 under certain treatment conditions such as using phage and honey at 50% (w/v) after different time points (see Figure 8). Based on this threshold and based on previous works reporting healing in ulcers only when the bacterial load was below 10^6^ CFU/ml (Bendy et al., 1964) and successful skin grafting in patients with wound contamination under 5 × 10^4^ CFU/cm^2^ (Majewski et al., 1995), it might be inferred that this combined treatment presents potential to effectively reduce viable bacterial levels. Moreover, the recognized anti-inflammatory activity of honey that stimulate immune responses by increasing the release of citokines supports this assumption (Visavadia et al., 2008).

In general, clear differences between the results obtained *in vitro* and *ex vivo* were observed. These include variations of viable cell reductions that can be mainly explained by the possible different biofilm architectures due to differences in the surfaces where the biofilms were formed, namely in roughness, and hydrophobicity, by the conditions adopted in each case that varied in terms of humidity and nutrient supply when biofilms were formed on polystyrene and porcine skin. Rougher materials tend, indeed, to promote bacterial adhesion due to microbial adherence to irregularities (Alnnasouri et al., 2011). In this work, *E. coli* showed 10-fold better ability to colonize the porcine skins than the polystyrene surfaces. Hydrophobicity can have an influence higher than roughness in surface colonization. In general, hydrophilic materials are favorable for cell attachment when bacteria have larger surface energy than the liquid in which they are suspended. However, the contrary is more common to happen, as bacterial surface energy is normally inferior to the surface energy of the liquids. This mismatch leads to cell adhesion preferentially to hydrophobic materials (Tuson and Weibel, 2013). According to Elkhyat et al. (2004) human skin contact angle is hydrophobic (91°) and therefore it is expected that porcine skins will also be hydrophobic. Polystyrene, on the other hand, is generally more hydrophilic than skin having a contact angle between 73° and 90° (Baier and Meyer, 1996; Cho et al., 2005; Kondyurin et al., 2006). The 10-fold higher colonization of porcine skin by *E. coli* suggests that the difference in surface roughness and hydrophobicity might be sufficient to interfere with the mechanisms of gene expression (including motility and attachment gene expression) (Tuson and Weibel, 2013), secretion of EPS, among other factors. These, particularly the secretion of EPS can have a great influence in the action of phages and honey, and even in the antimicrobial action of the individual components that are present in honeys.

Overall, this work provides novel insights into alternative strategies to control biofilm-related infections caused by *E. coli* and *P. aeruginosa* using phage-honey formulations. This work indicates that EC3a and PAO1-D may effectively be combined with chestnut honey to treat wound beds with *P. aeruginosa* and *E. coli* microbial biofilms. This formulation can potentially be used for topical applications due to the known advantages of phages in the control of antibiotic-resistant bacteria and of honeys ability to accelerate wound healing. Further improvements are required to obtain greater microbial reductions, which may include testing other phages and honey types as well as producing encapsulated particles where for instance phages are entrapped in the core and honey in the shell layer in order to preserve phage viability.

## Author contributions

AO and SS conceived the study. AS, JS, and LM performed the experiments. AO and SS wrote the paper. All authors critically analyzed and revised the manuscript.

### Conflict of interest statement

The authors declare that the research was conducted in the absence of any commercial or financial relationships that could be construed as a potential conflict of interest.

## References

[B1] Adams C. J. Boult C. H. Deadman B. J. Farr J. M. Grainger M. N. C. Manley-Harris M.. (2008). Isolation by HPLC and characterisation of the bioactive fraction of New Zealand manuka (Leptospermum scoparium) honey. Carbohydr. Res. 343, 651–659. 10.1016/j.carres.2007.12.011 18194804

[B2] Adams M. (1959). Bacteriophages. New York, NY: Interscience Publishers, Inc.

[B3] Agún S. Fernández L. González-Menéndez E. Martínez B. Rodríguez A. García P. (2018). Study of the interactions between bacteriophage phiIPLA-RODI and four chemical disinfectants for the elimination of *Staphylococcus aureus* contamination. Viruses 10:E103. 10.3390/v10030103 29495568 PMC5869496

[B4] Alnnasouri M. Lemaitrea C. Gentric C. Dagot C. Pon M.-N. (2011). Influence of surface topography on biofilm development: experiment and modeling. Biochem. Eng. J. 57, 38–45. 10.1016/j.bej.2011.08.005

[B5] Andrews J. M. Andrews J. M. (2001). Determination of minimum inhibitory concentrations. J. Antimicrob Chemother 48 (Suppl. 1), 5–16. 10.1093/jac/48.suppl_1.5 11420333

[B6] Baier R. E. Meyer A. E. (1996). Interfacial Phenomena and Bioproducts. New York, NY:Marcel Dekker.

[B7] Baillie G. S. Douglas L. J. (1998). Effect of growth rate on resistance of candida albicans biofilms to antifungal agents. Antimicrob. Agents Chemother. 42, 1900–1905. 9687381 10.1128/aac.42.8.1900 PMC105707

[B8] Bendy R. H. Nuccio P. A. Wolfe E. Collins B. Tamburro C. Glass W.. (1964). Counts to healing of decubiti: effect of topical gentamicin relationship of quantitative wound bacterial. Antimicrob. Agents Chemother. 10, 147–155. 14287920

[B9] Billings N. Ramirez Millan M. Caldara M. Rusconi R. Tarasova Y. Stocker R.. (2013). The extracellular matrix component Psl provides fast-acting antibiotic defense in *Pseudomonas aeruginosa* biofilms. PLoS Pathog. 9:e1003526. 10.1371/journal.ppat.1003526 23950711 PMC3738486

[B10] Bogdanov S. (2002). Harmonized Methods of the European Honey Commission. Swiss Bee Research Centre, FAM, Liebefeld.

[B11] Bogdanov S. Jurendic T. Sieber R. Gallmann P. (2008). Honey for nutrition and health: a review. J. Am. Coll. Nutr. 27, 677–689. 10.1080/07315724.2008.10719745 19155427

[B12] Bolognese F. Bistoletti M. Barbieri P. Orlandi V. T. (2016). Honey-sensitive *Pseudomonas aeruginosa* mutants are impaired in catalase A. Microbiol 162, 1554–1562. 10.1099/mic.0.000351 27516083

[B13] Bonaga G. Giumanini A. G. (1986). The volatile fraction of chestnut honey. J. Apic Res. 25, 113–120. 10.1080/00218839.1986.11100703

[B14] Bowler G. B. (2003). Bacterial growth guideline: reassessing its clinical relevance in wound healing. Ostomy Wound Manag. 49, 1–10. 12532033

[B15] Breitinger H.-G. (2012). Drug synergy – mechanisms and methods of analysis. Toxic Drug Test 143–166.

[B16] Brudzynski K. (2006). Effect of hydrogen peroxide on antibacterial activities of Canadian honeys. Can. J. Microbiol. 52, 1228–1237. 10.1139/w06-086 17473892

[B17] Cerqueira L. Oliveira J. A. Nicolau A. Azevedo N. F. Vieira M. J. (2013). Biofilm formation with mixed cultures of *Pseudomonas aeruginosa*/*Escherichia coli* on silicone using artificial urine to mimic urinary catheters. Biofouling 29, 829–840. 10.1080/08927014.2013.807913 23837894

[B18] Chaudhry W. N. Concepción-Acevedo J. Park T. Andleeb S. Bull J. J. Levin B. R.. (2017). Synergy and Order Effects of Antibiotics and Phages in Killing *Pseudomonas aeruginosa* Biofilms. PLoS ONE 12:e0168615. 10.1371/journal.pone.0168615 28076361 PMC5226664

[B19] Chiang W.-C. Nilsson M. Jensen P. O. Hoiby N. Nielsen T. E. Givskov M.. (2013). Extracellular DNA shields against aminoglycosides in *Pseudomonas aeruginosa* biofilms. Antimicrob. Agents Chemother. 57, 2352–2361. 10.1128/AAC.00001-13 23478967 PMC3632962

[B20] Cho J. S. Han S. Kim K. H. Han Y. G. Lee J. H. Lee C. S. (2005). Adhesion Aspects of Thin Films. Utrecht:VSP.

[B21] Cislo M. Dabrowski M. Weber-Dabrowska B. Woyton A. (1987). Bacteriophage treatment of suppurative skin infections. Arch. Immunol. Ther. Exp. 35, 175–183. 3447533

[B22] Coniglio M. A. Faro G. Giammanco G. Pignato S. Marranzano M. (2013). Antimicrobial potential of sicilian honeys against commensal *Escherichia coli* and pathogenic *Salmonella* serovar infantis. J. Prev. Med. Hyg. 54, 223–226. 24779285 PMC4718317

[B23] Crouzet M. Le Senechal C. Brözel V. S. Costaglioli P. Barthe C. Bonneu M.. (2014). Exploring early steps in biofilm formation: set-up of an experimental system for molecular studies. BMC Microbiol. 14:253. 10.1186/s12866-014-0253-z 25266973 PMC4189659

[B24] da Costa A. M. A. Machado R. Ribeiro A. Collins T. Thiagarajan V. Neves Petersen M. T.. (2015). Development of elastin like recombinamer films with antimicrobial activity. Biomacromolecules. 16, 625–635. 10.1021/bm5016706 25580615

[B25] Ding Y. Onodera Y. Lee J. C. Hooper D. C. (2008). NorB, an efflux pump in *Staphylococcus aureus* strain MW2, contributes to bacterial fitness in abscesses. J. Bacteriol. 190, 7123–7129. 10.1128/JB.00655-08 18723624 PMC2580682

[B26] Dunford C. E. Hanano R. (2004). Acceptability to patients of a honey dressing for non-healing venous leg ulcers. J. Wound Care 13, 193–197. 10.12968/jowc.2004.13.5.26614 15160574

[B27] Elkhyat A. Courderot-Masuyer C. Gharbi T. Humbert P. (2004). Influence of the hydrophobic and hydrophilic characteristics of sliding and slider surfaces on friction coefficient: *in vivo* human skin friction comparison. Ski Res. Technol. 10, 215–221. 10.1111/j.1600-0846.2004.00085.x 15536654

[B28] Ferraro M. J. Wikler M. A. Craig W. A. Dudley M. N. Eliopoulos G. M. Hecht D. W. (2003). Methods for Dilution Antimicrobial Susceptibility Tests for Bacteria That Grow Aerobically; Approved Standard, 6th Edn. Wayne, PA: Clinical and Laboratory Standards Institute.

[B29] Flemming H. Wingender J. (2010). The biofilm matrix. Nat. Rev. Microbiol. 8, 623–633. 10.1038/nrmicro2415 20676145

[B30] Fux C. A. Costerton J. W. Stewart P. S. Stoodley P. (2005). Survival strategies of infectious biofilms. Trends Microbiol. 13, 34–40. 10.1016/j.tim.2004.11.010 15639630

[B31] Gethin G. T. Cowman S. Conroy R. M. (2008). The impact of Manuka honey dressings on the surface pH of chronic wounds. Int. Wound J. 5, 185–194. 10.1111/j.1742-481X.2007.00424.x 18494624 PMC7951475

[B32] Gethin G. Cowman S. (2008). Bacteriological changes in sloughy venous leg ulcers treated with manuka honey or hydrogel: an RCT. J. Wound Care 17, 241–247. 10.12968/jowc.2008.17.6.29583 18666717

[B33] Greco W. Unkelbach H.-D. Pöch G. Sühnel J. Kundi M. W B (1992). Consensus on concepts and terminology for combined-action assessment: the Saariselkä Agreement. Arch. Complex Environ. Stud. 4, 65–69.

[B34] Hao J. J. Liu H. Donis-Gonzalez I. R. Lu X. H. Jones A. D. Fulbright D. W. (2012). Antimicrobial activity of chestnut extracts for potential use in managing soilborne plant pathogens. Plant Dis. 96, 354–360. 10.1094/PDIS-03-11-0169 30727136

[B35] Hengzhuang W. Ciofu O. Yang L. Wu H. Song Z. Oliver A.. (2013). High beta-lactamase levels change the pharmacodynamics of beta-lactam antibiotics in *Pseudomonas aeruginosa* biofilms. Antimicrob. Agents Chemother. 57, 196–204. 10.1128/AAC.01393-12 23089750 PMC3535908

[B36] Imran M. Hussain M. B. Baig M. (2015). A randomized, controlled clinical trial of honey-impregnated dressing for treating diabetic foot ulcer. J. Coll. Physicians Surg. Pak. 25, 721–725. 10.2015/JCPSP.721725 26454386

[B37] Ito A. Taniuchi A. May T. Kawata K. Okabe S. (2009). Increased antibiotic resistance of Escherichia coli in mature biofilms. Appl. Environ. Microbiol. 75, 4093–4100. 10.1128/AEM.02949-08 19376922 PMC2698376

[B38] Jensen P. O. Bjarnsholt T. Phipps R. Rasmussen T. B. Calum H. Christoffersen L.. (2007). Rapid necrotic killing of polymorphonuclear leukocytes is caused by quorum-sensing-controlled production of rhamnolipid by *Pseudomonas aeruginosa*. Microbiology 153, 1329–1338. 10.1099/mic.0.2006/003863-0 17464047

[B39] Kilty S. J. Duval M. Chan F. T. Ferris W. Slinger R. (2011). Methylglyoxal: (Active agent of manuka honey) in vitro activity against bacterial biofilms. Int. Forum Allergy Rhinol. 1, 348–350. 10.1002/alr.20073 22287464

[B40] Kondyurin A. Gan B. K. Bile M. M. M. Mizuno K. McKenzie D. R. (2006). Etching and structural changes of polystyrene films during plasma immersion ion implantation from argon plasma. Nucl Instrum. Methods Phys Res Sect B 251, 413–418. 10.1016/j.nimb.2006.06.027

[B41] Lazarus G. S. Cooper D. M. Knighton D. R. Percoraro R. E. Rodeheaver G. Robson M. C. (1994). Definitions and guidelines for assessment of wounds and evaluation of healing. Wound Repair Regen. 2, 165–170. 10.1046/j.1524-475X.1994.20305.x 17156107

[B42] Lee J. H. Park J. H. Kim J. A. Neupane G. P. Cho M. H. Lee C. S.. (2011). Low concentrations of honey reduce biofilm formation, quorum sensing, and virulence in *Escherichia coli* O157:H7. Biofouling 27, 1095–1104. 10.1080/08927014.2011.633704 22047137

[B43] Lu J. Carter D. A. Turnbull L. Rosendale D. Hedderley D. Stephens J.. (2013). The effect of new zealand kanuka, manuka and clover honeys on bacterial growth dynamics and cellular morphology varies according to the species. PLoS ONE 8:e0055898. 10.1371/journal.pone.0055898 23418472 PMC3572166

[B44] Lu J. Turnbull L. Burke C. M. Liu M. Carter D. a Schlothauer R. C.. (2014). Manuka-type honeys can eradicate biofilms produced by *Staphylococcus aureus* strains with different biofilm-forming abilities. PeerJ 2:e326. 10.7717/peerj.326 24711974 PMC3970805

[B45] Majewski W. Cybulski Z. Napierala M. Pukacki F. Staniszewski R. Pietkiewicz K.. (1995). The value of quantitative bacteriological investigations in the monitoring of treatment of ischaemic ulcerations of lower legs. Int. Angiol. 14, 381–384. 8708431

[B46] Majtan J. (2014). Honey: an immunomodulator in wound healing. Wound Repair Regen. 22, 187–192. 10.1111/wrr.12117 24612472

[B47] Majtan J. Bohova J. Prochazka E. Klaudiny J. (2014). Methylglyoxal may affect hydrogen peroxide accumulation in manuka honey through the inhibition of glucose oxidase. J. Med. Food 17, 290–293. 10.1089/jmf.2012.0201 24192110 PMC3929242

[B48] Markoishvili K. Tsitlanadze G. Katsarava R. Morris J. G. J. Sulakvelidze A. (2002). A novel sustained-release matrix based on biodegradable poly(ester amide)s and impregnated with bacteriophages and an antibiotic shows promise in management of infected venous stasis ulcers and other poorly healing wounds. Int. J. Dermatol. 41, 453–458. 10.1046/j.1365-4362.2002.01451.x 12121566

[B49] Mendes J. J. Leandro C. Corte-Real S. Barbosa R. Cavaco-Silva P. Melo-Cristino J.. (2013). Wound healing potential of topical bacteriophage therapy on diabetic cutaneous wounds. Wound Repair Regen. 21, 595–603. 10.1111/wrr.12056 23755910

[B50] Mohamed H. (2017). Healing of chronic diabetic foot ulcers with natural honey: an alternative paradigm in wound healing. Wound Repair Regen. 25:A18. 10.1111/wrr.1257324843434

[B51] Molan P. C. (1992). The Anitbacterial Activity of Honey: 1. The nature of the antibacterial activity. Bee World 73, 5–28.

[B52] Molan P. C. Betts J. A. (2004). Clinical usage of honey as a wound dressing: an update. J. Wound Care 13, 353–356. 10.12968/jowc.2004.13.9.26708 15517742

[B53] Mustoe T. A. O'Shaughnessy K. Kloeters O. (2006). Chronic wound pathogenesis and current treatment strategies: a unifying hypothesis. Plast Reconstr. Surg. 117, 35S−41S. 10.1097/01.prs.0000225431.63010.1b 16799373

[B54] Naghili H. Tajik H. Mardani K. Razavi Rouhani S. M. Ehsani A. Zare P. (2013). Validation of drop plate technique for bacterial enumeration by parametric and nonparametric tests. Vet Res forum an Int Q J 4, 179–183. 25653794 PMC4312378

[B55] Nishio E. K. Ribeiro J. M. Oliveira A. G. Andrade C. G. T. J. Proni E. A. Kobayashi R. K. T.. (2016). Antibacterial synergic effect of honey from two stingless bees: *Scaptotrigona bipunctata* Lepeletier, 1836, and S. postica Latreille, (1807). Sci. Rep. 6:21641. 10.1038/srep21641 26869239 PMC4751499

[B56] Oryan A. Alemzadeh E Moshiri A. (2016). Biological properties and therapeutic activities of honey in wound healing: a narrative review and meta-analysis. J. Tissue Viability 25, 98–118. 10.1016/j.jtv.2015.12.002 26852154

[B57] Oliveira A. Ribeiro H. G. Silva A. C. Silva M. D. Sousa J. C. Rodrigues C. F.. (2017). Synergistic antimicrobial interaction between honey and phage against *Escherichia coli* biofilms. Front. Microbiol. 8:2407. 10.3389/fmicb.2017.02407 29276503 PMC5727068

[B58] Park D. J. Drobniewski F. A. Meyer A. Wilson S. M. (2003). Use of a phage-based assay for phenotypic detection of mycobacteria directly from sputum. J. Clin. Microbiol. 41, 680–688. 10.1128/JCM.41.2.680-688.2003 12574267 PMC149652

[B59] Piggott J. J. Townsend C. R. Matthaei C. D. (2015). Reconceptualizing synergism and antagonism among multiple stressors. Ecol. Evol. 5, 1538–1547. 10.1002/ece3.1465 25897392 PMC4395182

[B60] Pires D. P. Dötsch A. Anderson E. M. Hao Y. Khursigara C. M. Lam J. S.. (2017). A genotypic analysis of five *P. aeruginosa* strains after biofilm infection by phages targeting different cell surface receptors. Front. Microbiol. 8:1229. 10.3389/fmicb.2017.01229 28713356 PMC5492357

[B61] Pires D. P. Kropinski A. M. Azeredo J. Sillankorva S. (2014). Complete genome sequence of the *Pseudomonas aeruginosa* Bacteriophage phiIBB-PAA2. Genome Announc. 2, e01102–e01113. 10.1128/genomeA.01102-13 24503992 PMC3916486

[B62] Pires D.P. Sillankorva S. Faustino A. Azeredo J. (2011). Use of newly isolated phages for the control of *Pseudomonas aeruginosa* PAO1 and ATCC 10145 biofilms. Res. Microbiol. 162, 798–806. 10.1016/j.resmic.2011.06.010 21782936

[B63] Pires D. P. Sillankorva S. Kropinski A. M. Lu T. K. Azeredo J. (2015). Complete Genome Sequence of *Pseudomonas aeruginosa* Phage vB_PaeM_CEB_DP1. Genome Announc. 3:e00918. 10.1128/genomeA.00918-15 26404589 PMC4582565

[B64] Pirnay J. P. De Vos D. Verbeken G. Merabishvili M. Chanishvili N. Vaneechoutte M.. (2011). The phage therapy paradigm: Prêt-à-porter or sur-mesure? Pharm. Res. 28, 934–937. 10.1007/s11095-010-0313-5 21063753

[B65] Robson M. C. (1997). Wound infection: a failure of wound healing caused by an imbalance of bacteria. Surg. Clin. North Am. 77, 637–650. 10.1016/S0039-6109(05)70572-7 9194884

[B66] Rossano R. Larocca M. Polito T. Perna A. M. Padula M. C. Martelli G.. (2012). What are the proteolytic enzymes of honey and what they do tell us? a fingerprint analysis by 2-D Zymography of Unifloral Honeys. PLoS ONE 7:e0049164. 10.1371/journal.pone.0049164 23145107 PMC3492327

[B67] Sambrook J. W. Russell D. (2001). Molecular Cloning: A Laboratory Manual. New York, NY: Cold Spring Harb Lab Press.

[B68] Sato T. Miyata G. (2000). The nutraceutical benefit, Part III: Honey. Nutrition 16, 468–469. 10.1016/S0899-9007(00)00271-9 10869910

[B69] Seth A. K. Geringer M. R. Nguyen K. T. Agnew S. P. Dumanian Z. Galiano R. D.. (2013). Bacteriophage therapy for *Staphylococcus aureus* biofilm-infected wounds: a new approach to chronic wound care. Plast. Reconstr. Surg. 131, 225–234. 10.1097/PRS.0b013e31827e47cd 23357984

[B70] Sharp A. (2009). Beneficial effects of honey dressings in wound management. Nurs Stand. 24, 66–68. 10.7748/ns.24.7.66.s54 19927561

[B71] Sillankorva S. Neubauer P. Azeredo J. (2008). Isolation and characterization of a T7-like lytic phage for *Pseudomonas fluorescens*. BMC Biotechnol. 8:80. 10.1186/1472-6750-8-80 18954452 PMC2582237

[B72] Tuson H. H. Weibel D. B. (2013). Bacteria-surface interactions. Soft. Matter. 9, 4368–4380. 10.1039/c3sm27705d 23930134 PMC3733390

[B73] United States Deparment of Agriculture (1985). United States Standards for Grades of Extracted Honey. Fed. Regist. 50FR15861:R15812.

[B74] Visavadia B. G. Honeysett J. Danford M. H. (2008). Manuka honey dressing: an effective treatment for chronic wound infections. Br. J. Oral Maxillofac. Surg. 46, 55–56. 10.1016/j.bjoms.2006.09.013 17113690

[B75] Voncina B. Zemljič Fras L. Ristic T. (2015). Active Textile Dressings for Wound Healing. in Advances in Smart Medical Textiles: Treatments and Health Monitoring, ed Langenhove L. (Swaston; Cambridge, MA; Woodhead Publishing), 73–92.

[B76] Weinbauer M. G. (2004). Ecology of prokaryotic viruses. FEMS Microbiol. Rev. 28, 127–181. 10.1016/j.femsre.2003.08.001 15109783

[B77] White J. W. (1979). Spectrophotometric method for hydroxymethylfurfural in honey. J. Assoc. 62, 509–514. 479072

[B78] Whiteley M. Bangera M. G. Bumgarner R. E. Parsek M. R. Teitzel G. M. Lory S.. (2001). Gene expression in *Pseudomonas aeruginosa* biofilms. Nature 413, 860–864. 10.1038/35101627 11677611

[B79] Zhang L. Mah T.-F. (2008). Involvement of a novel efflux system in biofilm-specific resistance to antibiotics. J. Bacteriol. 190, 4447–4452. 10.1128/JB.01655-07 18469108 PMC2446775

