# Supplementary material for: Chestnut Honey and Bacteriophage Application to Control Pseudomonas aeruginosa and Escherichia coli Biofilms: Evaluation in an ex vivo Wound Model
Source: Front Microbiol. 2018 Jul 31;9:1725. doi: 10.3389/fmicb.2018.01725 (PMC6080586; doi:10.3389/fmicb.2018.01725)
Supplement: Supplementary file 1 [file Data_Sheet_1.docx]

**Table S1.** Host-range of isolated phages against P. aeruginosa clinical isolates and reference strains.

|  | Susceptibility to phage * | | | | | | |
| --- | --- | --- | --- | --- | --- | --- | --- |
| Isolate | PAO1-A | PAO1-B | PAO1-C | PAO1-D | PAO1-E | PAO1-F | PAO1-G |
| Pa 1 | - | - | - | - | +- | - | - |
| Pa 2 | - | - | - | - | +- | + | - |
| Pa 3 | +- | +- | +- | +- | + | +- | +- |
| Pa 4 | +- | + | + | + | + | + | + |
| Pa 5 | +- | +- | +- | +- | + | + | +- |
| Pa 6 | +- | +- | +- | + | + | + | +- |
| Pa 7 | +- | + | + | + | + | + | + |
| Pa 8 | +- | +- | +- | + | + | +- | + |
| Pa 9 | + | + | + | + | + | + | + |
| Pa 10 | - | - | - | - | - | - | - |
| Pa 11 | - | - | - | - | - | - | - |
| Pa 12 | +- | +- | + | + | + | +- | + |
| Pa 14 | +- | +- | +- | +- | +- | +- | +- |
| Pa 15 | + | + | + | + | + | + | + |
| Pa 16 | - | - | - | - | - | - | - |
| Pa 17 | - | - | - | +- | +- | - | +- |
| Pa 18 | + | + | +- | +- | - | +- | +- |
| Pa 19 | + | + | + | + | + | + | + |
| Pa 20 | +- | +- | +- | +- | +- | +- | +- |
| Pa 21 | - | - | - | - | - | - | - |
| Pa 22 | - | - | - | - | - | - | - |
| Pa 23 | +- | +- | +- | + | + | + | + |
| Pa 24 | - | - | - | - | - | - | - |
| Pa 25 | - | - | - | - | - | - | - |
| Pa 26 | - | - | - | - | - | - | - |
| Pa 27 | - | - | - | - | - | - | - |
| Pa 28 | - | - | - | - | - | - | - |
| Pa 29 | - | - | - | - | - | - | - |
| Pa S20 | + | + | +- | + | + | + | + |
| Pa S21 | + | + | +- | + | + | + | + |
| Pa S22 | + | + | +- | + | + | + | + |
| Pa S23 | - | - | - | +- | +- | +- | - |
| Pa S24 | - | - | - | - | - | - | +- |
| Ref CECT III | + | + | +- | + | + | + | + |
| Ref PAO 1 | + | + | + | + | + | + | + |
| Ref ATCC 10145 | + | + | +- | + | + | + | +- |
| Sum of “+” | 10 | 12 | 7 | 15 | 17 | 15 | 13 |

* “+” completely lysed – no phage resistance colonies were observed; “+-” partially lysed – phage resistant colonies observed; “-” no lysis.

**Table S2.** Determination of phage plaque and halo dimensions for the seven *P. aeruginosa* phages isolated from two commercial phage cocktails. Data are shown as mean ± SD


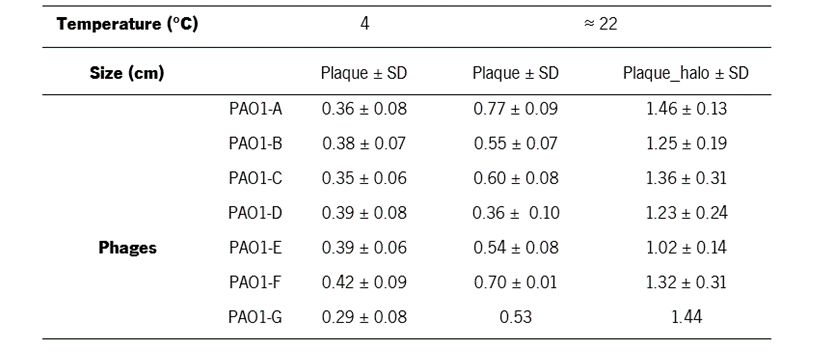


Table S3. Determination of possibility of synergism occurrence in combined therapy experiments.

| *Dual-species biofilms formed in vitro – results for E. coli 434* | | |
| --- | --- | --- |
| Time (h) | Log(C)-log(S_P_)-log(S_H25%_)+log(S_PH25%_) | Log(C)-log(S_P_)-log(S_H50%_)+log(S_PH50%_) |
| 6 | 0.53 | 0.58 |
| 12 | 1.15 | 0.67 |
| 24 | 1.08 | 1.48 |
| *Dual-species biofilms formed in vitro – results for P. aeruginosa PAO1* | | |
| Time (h) | Log(C)-log(S_P_)-log(S_H25%_)+log(S_PH25%_) | Log(C)-log(S_P_)-log(S_H50%_)+log(S_PH50%_) |
| 6 | 0.15 | 0.70 |
| 12 | 0.30 | 1.04 |
| 24 | 0.89 | 2.05 |
| *Monospecies E. coli biofilms formed ex vivo* | | |
| Time (h) | Log(C)-log(S_P_)-log(S_H25%_)+log(S_PH25%_) | Log(C)-log(S_P_)-log(S_H50%_)+log(S_PH50%_) |
| 6 | 0.24 | 1.30 |
| 12 | 0.27 | 0.74 |
| 24 | 1.02 | 0.82 |
| *Monospecies P. aeruginosa biofilms formed ex vivo* | | |
| Time (h) | Log(C)-log(S_P_)-log(S_H25%_)+log(S_PH25%_) | Log(C)-log(S_P_)-log(S_H50%_)+log(S_PH50%_) |
| 6 | -2.72 | -0.11 |
| 12 | -1.00 | -0.69 |
| 24 | 0.95 | 1.07 |
| *Dual-species biofilms formed ex vivo – results for E. coli 434* | | |
| Time (h) | Log(C)-log(S_P_)-log(S_H25%_)+log(S_PH25%_) | Log(C)-log(S_P_)-log(S_H50%_)+log(S_PH50%_) |
| 6 | -0.36 | -0.09 |
| 12 | 0.61 | 0.78 |
| 24 | -0.10 | -0.19 |
| *Dual-species biofilms formed ex vivo – results for P. aeruginosa PAO1* | | |
| Time (h) | Log(C)-log(S_P_)-log(S_H25%_)+log(S_PH25%_) | Log(C)-log(S_P_)-log(S_H50%_)+log(S_PH50%_) |
| 6 | -0.59 | -0.54 |
| 12 | 0.07 | -1.10 |
| 24 | -0.79 | 1.24 |

Grey coloured cells indicate values where synergy outcome was reached.


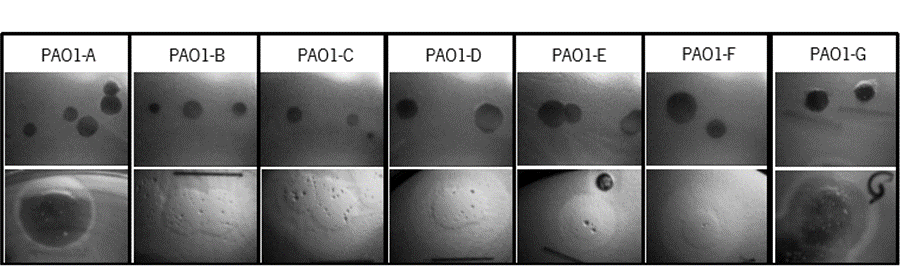


**Figure S1**. Microscopic observation of plaques and halos of *P. aeruginosa* phages, after incubation at 37 ºC overnight. Top line displays phages stored at 4 ºC and bottom line shows phages and their halos stored for 3 days at room temperature. Black bar represents 1 cm.
